# P14 Challenges in treating anti-melanoma differentiation associated gene 5 (MDA5) antibody positive juvenile dermatomyositis (JDM) with interstitial lung disease (ILD) complicated by pneumocytis jirovecii pneumonia (PCP): a case report

**DOI:** 10.1093/rap/rkac067.014

**Published:** 2022-09-28

**Authors:** Sharifah Adrianna Affendi, Emma Jackson, Muthana Al-Obaidi

**Affiliations:** Great Ormond Street Hospital for Children, London, United Kingdom; University of Cambridge, Cambridge, United Kingdom; Great Ormond Street Hospital for Children, London, United Kingdom

## Abstract

**Introduction/Background:**

We describe a case of MDA5 antibody positive JDM associated with ILD who developed worsening in breathing effort requiring oxygen support. We highlight the challenges associated with the risk of rapidly progressing ILD (RPILD) versus infection as the patient deteriorated when her oral prednisolone was tapered. Whilst investigating for other potential causes of RPILD, immunosuppression was intensified. A suboptimal response to treatment prompted extensive opportunistic infection screening identifying pneumocystis pneumonia (PCP). Here, we describe the clinical course of this patient, outline the management, and ask whether immunosuppressed JDM patients with ILD should be prophylactically treated for fungal disease.

**Description/Method:**

A 12-year-old girl presented with a 4 months history of oral ulcers, exercise intolerance, joint stiffness, and erythematous palmar rash. On examination, she had a weak swallowing with swelling and restriction in her joints, Gottron’s papules, vasculitic rash with skin ulceration and dilated nailfold capillaries. Manual muscle testing (MMT)-8 and Childhood Muscle Assessment Scale (CMAS) scores were 54/80 and 42/52 respectively. Based on this clinical presentation and further tests she was diagnosed with MDA5 positive JDM.

She developed symptoms suspicious of pulmonary embolism, prompting a CT pulmonary angiogram. This identified coincidental scattered areas of ground-glass consolidation with atelectasis consistent with ILD. Lung function testing (LFT) was normal (FEV1 2.89L (100.2%), FVC 2.94L (89.9%), DLCO 87%, KCO 98.4%).

She was treated with pulses of IV methylprednisolone (IVMP), IV immunoglobulin (IVIG), methotrexate, and rituximab. Methotrexate was stopped as she developed transaminitis and switched to ciclosporin. She was discharged with prednisolone and ciclosporin.

She presented 6 weeks later with dyspnoea, fatigue and new skin rashes, but had no cough. Examination found reduced breath sounds and crackles.

Investigations showed CRP <5, ESR 80mm/hour, WCC 15.45 (x10*9/L), ALT 43 (U/L), AST 80 (U/L), CK 21 (U/L), LDH 639 (U/L). Chest radiograph on admission was reported as normal and CT thorax showed non-specific patchy mild ground-glass opacification. LFT showed worsening restrictive picture with decreased diffusing capacity with FEV1 1.56L (53%), FVC 1.79L (54%), DLCO 46%, and KCO 73%. Progression of ILD was suspected thus she was pulsed with IVMP and given IV cyclophosphamide.

Fever with worsening respiratory distress ensued requiring PICU admission, prompting an extensive infection screen. PCP PCR and β -D glucan were positive, thus she was treated for PCP with co-trimoxazole and caspofungin. She made good recovery and was discharged well.

**Discussion/Results:**

Juvenile dermatomyositis (JDM) is a rare systemic autoimmune condition in childhood. It primarily affects the proximal muscles and skin but may also affect the heart, respiratory, GI system, joint and other organs. It is a complex condition and is potentially life threatening if treated inadequately. The emergence of myositis specific antibodies (MSA) have enhanced our understanding of both the clinical and laboratory features of JDM. The phenotype of children with anti-MDA5 autoantibodies has yet to be clearly established unlike their adult counterpart however there have been increased recognition that it is associated with risk of developing ILD. In our case, MDA5-associated ILD was diagnosed at presentation and she was treated aggressively with immunosuppressants. She developed deterioration in her respiratory symptoms 6 weeks later whilst on weaning course of steroids triggering the possibility of worsening ILD. This has led to her receiving a course of intravenous cyclophosphamide in addition to on-going immunosuppressants. However, as she worsened despite optimal treatment for ILD, she was screened for opportunistic infections and was positive for PCP. This highlights challenges faced by clinicians in identifying the cause of worsening respiratory function in a children with established ILD who are immunosuppressed. It maybe multi-factorial however opportunistic infections such as PCP should always be considered in these children to ensure it is treated appropriately to prevent further complications.

**Key learning points/Conclusion:**

Anti-MDA5 positive ILD represents a cohort of children requiring a more aggressive immunosuppressive therapies to optimise and prevent further deterioration in their clinical respiratory function. However, since there are no proven biomarkers able to differentiate indolent ILD from RPILD, it may be favourable to assume RPILD and treat aggressively.

Balancing the risks and benefits caused by aggressive immunosuppression in these children continues to be a challenge. Therefore, clinicians should be cognisant of opportunistic infections such as PCP when faced with children with underlying ILD whose respiratory function deteriorates. Chemoprophylaxis with co-trimoxazole is generally well tolerated with minimal side effects and therefore should be considered in these children at diagnosis.

